# Dynamic changes in circulating T follicular helper cell composition predict neutralising antibody responses after yellow fever vaccination

**DOI:** 10.1002/cti2.1129

**Published:** 2020-05-13

**Authors:** Johanna E Huber, Julia Ahlfeld, Magdalena K Scheck, Magdalena Zaucha, Klaus Witter, Lisa Lehmann, Hadi Karimzadeh, Michael Pritsch, Michael Hoelscher, Frank von Sonnenburg, Andrea Dick, Giovanna Barba‐Spaeth, Anne B Krug, Simon Rothenfußer, Dirk Baumjohann

**Affiliations:** ^1^ Institute for Immunology Biomedical Center Faculty of Medicine LMU Munich Planegg‐Martinsried Germany; ^2^ Division of Clinical Pharmacology University Hospital LMU Munich Munich Germany; ^3^ Einheit für Klinische Pharmakologie (EKLiP) Helmholtz Zentrum München German Research Center for Environmental Health (HMGU) Neuherberg Germany; ^4^ Laboratory of Immunogenetics and Molecular Diagnostics Department of Transfusion Medicine, Cell Therapeutic Agents and Hemostaseology LMU Munich Munich Germany; ^5^ Division of Infectious Diseases and Tropical Medicine University Hospital LMU Munich Munich Germany; ^6^ German Center for Infection Research, partner site Munich Munich Germany; ^7^ Structural Virology Unit and CNRS UMR 3569 Virology Department Institut Pasteur Paris France; ^8^ Medical Clinic III for Oncology, Hematology, Immuno‐Oncology and Rheumatology University Hospital Bonn University of Bonn Bonn Germany; ^9^ Present address: Department of Pharmacy LMU Munich Munich Germany

**Keywords:** neutralising antibodies, T follicular helper (Tfh) cells, vaccination, viral infection, yellow fever, YF‐17D

## Abstract

**Objectives:**

T follicular helper (Tfh) cells are the principal T helper cell subset that provides help to B cells for potent antibody responses against various pathogens. In this study, we took advantage of the live‐attenuated yellow fever virus (YFV) vaccine strain, YF‐17D, as a model system for studying human antiviral immune responses *in vivo* following exposure to an acute primary virus challenge under safe and highly controlled conditions, to comprehensively analyse the dynamics of circulating Tfh (cTfh) cells.

**Methods:**

We tracked and analysed the response of cTfh and other T and B cell subsets in peripheral blood of healthy volunteers by flow cytometry over the course of 4 weeks after YF‐17D vaccination.

**Results:**

Using surface staining of cell activation markers to track YFV‐specific T cells, we found increasing cTfh cell frequencies starting at day 3 and peaking around 2 weeks after YF‐17D vaccination. This kinetic was confirmed in a subgroup of donors using MHC multimer staining for four known MHC class II epitopes of YF‐17D. The subset composition of cTfh cells changed dynamically during the course of the immune response and was dominated by the cTfh1‐polarised subpopulation. Importantly, frequencies of cTfh1 cells correlated with the strength of the neutralising antibody response, whereas frequencies of cTfh17 cells were inversely correlated.

**Conclusion:**

In summary, we describe detailed cTfh kinetics during YF‐17D vaccination. Our results suggest that cTfh expansion and polarisation can serve as a prognostic marker for vaccine success. These insights may be leveraged in the future to improve current vaccine design and strategies.

## Graphical abstract

We tracked circulating T follicular helper (cTfh) cells in the blood of healthy individuals who received the yellow fever (YF) vaccine YF‐17D. We found that cTfh1‐polarised cells dominated the cTfh response and that their frequency on day 14 predicted YF virus‐neutralising antibody levels detected on day 28 after vaccination, thus highlighting the prognostic value of cTfh cells for monitoring of vaccine outcomes.

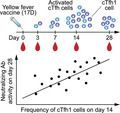

## Introduction

Emerging new viral infections and re‐emerging known viral pathogens are posing an ever‐growing threat, especially if preventive measures in form of vaccinations are not available or limited. Recent outbreaks of Zika, Ebola and yellow fever virus (YFV) in the tropics^1^ as well as measles and yearly influenza waves in Europe and the United States, and the current worldwide spread of the new coronavirus SARS‐CoV‐2 that causes COVID‐19, serve as a reminder to the public and health authorities that prevention and treatment of viral diseases remain one of the biggest medical challenges. Thus, a solid understanding of the cellular immune response to viral infection is of immense importance for the development of new vaccines and antiviral treatments.

One of the most potent and successful vaccines in current use is the vaccination against YFV. The live‐attenuated YFV strain 17D has been developed and used since the 1930s,^2^ leading to livelong immunity in over 98% of vaccinees after a single shot.^3^ This efficiency relies on the generation of neutralising antibodies as well as on the formation of CD4^+^ T cell memory.^4^, ^5^, ^6^ Neutralising antibodies are detected starting 10 days post‐vaccination and can persist for decades after immunisation.^7^ Adverse events of the vaccination are extremely rare, which makes the vaccination with YF‐17D a safe and beneficial intervention that can be used to model an acute viral infection to study innate and adaptive antiviral immune responses in human subjects.

Production of high and long‐lasting titres of neutralising antibodies suggests involvement of germinal centres (GCs). During a GC reaction, B cells undergo class‐switching and affinity maturation and rely on help from a specific subset of CD4^+^ T cells, the so‐called T follicular helper (Tfh) cells.^8^, ^9^, ^10^ The GC reaction is generally difficult to study in humans as it occurs in secondary lymphoid organs (such as lymph nodes), which are difficult to sample. However, CD4^+^ T cells expressing the Tfh subset‐defining chemokine receptor CXCR5 can also be found in peripheral blood and have therefore been named circulating T follicular helper (cTfh) cells.^11^ cTfh cells represent the circulating memory compartment of human Tfh cells that are clonally related to GC Tfh cells.^12^, ^13^, ^14^ cTfh cells can perform classical Tfh tasks such as promoting class‐switching in B cells and antibody production and they have been described to promote vaccine‐induced immunity.^15^, ^16^ Similar to classical T helper cell subsets, cTfh cells can be divided into subsets (cTfh1, cTfh2, cTfh17, cTfh‐1‐17) according to the expression of the chemokine receptors CXCR3 and CCR6.^11^

Circulating T follicular helper cells are therefore considered a promising target to improve vaccination strategies.^17^ While the CD8^+^ T cell response to YFV has been described in detail previously,^18^, ^19^, ^20^ the CD4^+^ T cell response has only recently drawn attention.^21^ Importantly, cTfh cells have not been investigated to date in detail in the immune response following YF‐17D vaccination. The extraordinary neutralising antibody response induced by YF‐17D is not yet fully understood, but in experiments performed in animal models, it was shown that the co‐transfer of immune sera and CD4^+^ T cells provided complete protection, whereas transfer of CD4^+^ T cells or immune sera alone only lead to partial protection.^4^ These data suggest that coordinated cellular and humoral immune responses are critical for mounting protective immune responses. In this study we used the YF‐17D vaccination model to assess and carefully characterise the frequencies and phenotypic changes of cTfh cells in healthy individuals after an acute viral infection.

## Results

### Activated circulating T follicular helper cells accumulate in the blood following YF‐17D vaccination

**Figure 1:**
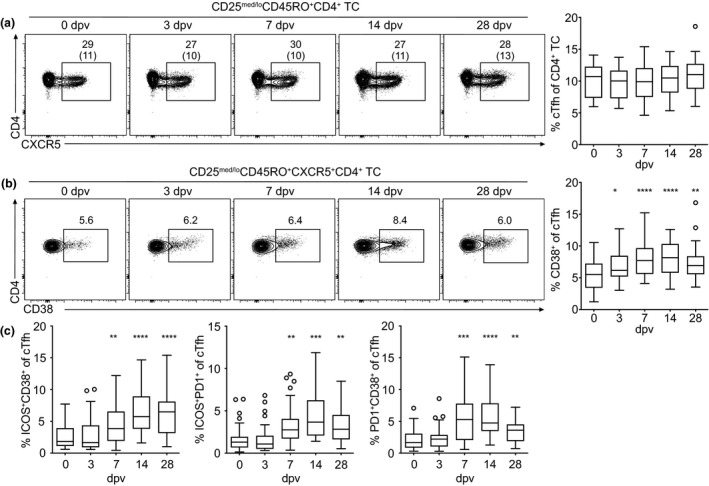
Circulating Tfh cells upregulate activation markers following YF‐17D vaccination. **(a–c)** PBMCs isolated before (day 0) and at the indicated time points after YF‐17D vaccination were analysed by flow cytometry (see Supplementary figure 1 for the gating strategy). Representative contour plots and quantification of **(a)** cTfh frequency and **(b, c)** cTfh activation determined by CD38, ICOS and PD‐1 expression after yellow fever vaccination are shown. Gate frequencies indicate the frequency in regard to the parent gate. Gate frequencies in brackets indicate the frequency of the population in regard to the reference population as indicated above. **(a–c)** Frequencies of Tfh cells and their activation status pooled from four independent experiments with 5 to 10 study participants each are presented as Tukey boxplots showing the median with the 25th and 75th percentile (*n* = 33) and whiskers and outliers calculated as highest and lowest observation below/above 1.5 times interquartile range. Data points below/above 1.5 times interquartile range are displayed individually. Statistical analysis was performed using repeated‐measure (RM) one‐way ANOVA and Dunnett's multiple comparison analysis to compare indicated time points to day 0. *P < 0.0332, **P < 0.0021, ***P < 0.0002, ****P < 0.0001. dpv, day(s) post‐vaccination.

Here, we used the YF‐17D vaccination model to study adaptive immune cell dynamics during an acute viral infection in healthy individuals, with a particular focus on Tfh cells using flow cytometry (see Supplementary figure 1 for gating strategies and definition of lymphocyte subpopulations). YF‐17D vaccination lead to a characteristic transient decrease in the absolute cell numbers of CD8^+^ cells on day 3 and day 7 after vaccination and a significant increase in the frequency of CD38‐expressing activated CD8^+^ T cells from day 3 on, peaking on day 14 (Supplementary figure 2a and b), which is in line with previous reports.^21^, ^22^ We also observed a small but non‐significant drop of total CD4^+^ T cell numbers and no change in the overall frequency of CD45RO^+^ memory CD4^+^ T cells (Supplementary figure 3a and b). While the frequency of total CXCR5^+^ cTfh cells did not change significantly during the immune response to the YFV over the time course of 28 days after vaccination, the absolute number of cTfh cells was slightly decreased on day 3 and day 7 (Figure 1a, Supplementary figure 3c). In contrast, we observed an increase in the frequency of activated CD38^+^ cTfh cells from day 3 post‐vaccination on and a further increase until day 7 and day 14, followed by a contraction thereafter, however, without completely reaching pre‐vaccination levels by day 28 (Figure 1b). Besides CD38, ICOS and PD‐1 have also been previously described as activation markers expressed by antigen‐specific cTfh cells.^14^, ^23^, ^24^, ^25^ Frequencies of cTfh cells co‐expressing either combination of these three markers were also increased significantly from day 7 on through day 28 (Figure 1c).

### The cTfh response to YF‐17D vaccination is dominated by the cTfh1 subset

**Figure 2:**
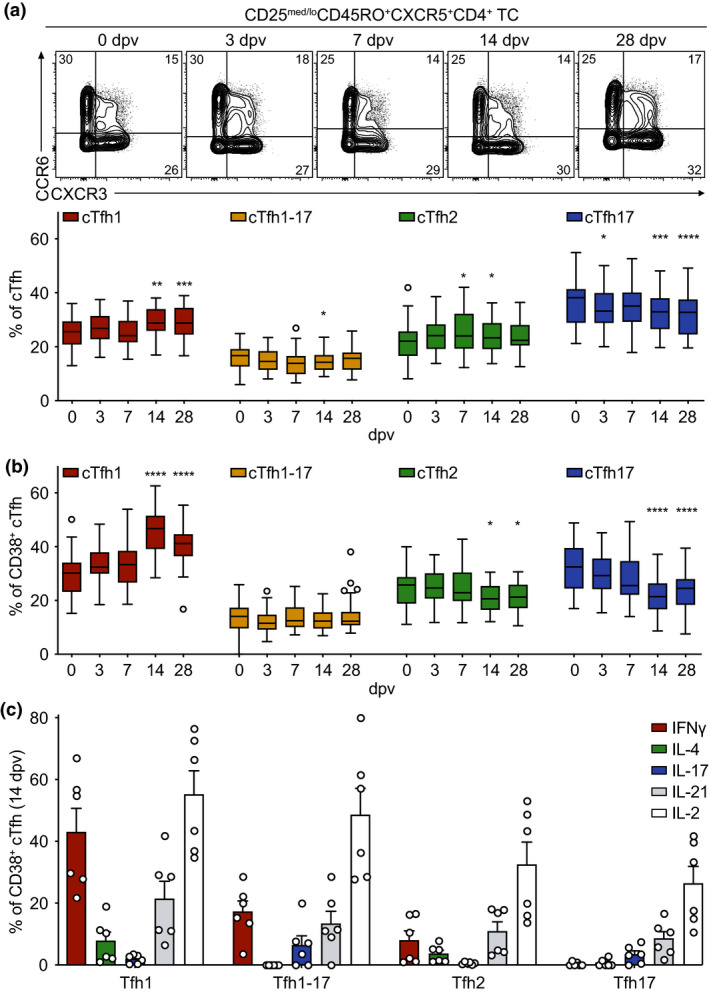
Changes in the polarisation of circulating Tfh cells after yellow fever vaccination **(a, b)** PBMCs isolated before (day 0) and at the indicated time points after YF‐17D vaccination were analysed by flow cytometry (see Supplementary figure 1 for the gating strategy). Representative contour plots and quantification of frequencies of **(a)** cTfh cell subsets and **(b)** activated CD38^+^ cTfh cell subsets identified according to CXCR3 and CCR6 expression are shown for the indicated time points. **(c)** Frequencies of cytokine‐expressing CD38^+^ cTfh cells were measured by intracellular antibody staining after re‐stimulation *ex vivo* with PMA/ionomycin on day 14 after YF‐17D vaccination. **(a, b)** Pooled data from four independent experiments with 5 to 10 study participants each are presented as Tukey boxplots showing the median with the 25th and 75th percentile (*n* = 33) and whiskers and outliers calculated as highest and lowest observation below/above 1.5 times interquartile range. Data points below/above 1.5 times interquartile range are displayed individually. Statistical analysis was performed using RM one‐way ANOVA and Dunnett's multiple comparison analysis to compare indicated time points to day 0. *P < 0.0332, **P < 0.0021, ***P < 0.0002, ****P < 0.0001. **(c)** Representative data of one of four separately performed experiments are shown as mean and SEM, with each dot representing one donor (*n* = 6).

The peak of activation on day 14 was accompanied by a change in the polarisation of cTfh cells compared to the pre‐vaccination state, which can be analysed by assessment of different cTfh cell subsets defined by their CXCR3 and CCR6 expression (gating strategy in Supplementary figure 1a).^11^ CXCR3^+^CCR6^−^ cTfh1 cells were increased in frequency on day 14 and day 28, whereas frequencies of CXCR3^−^CCR6^+^ cTfh17 cells were decreased on those days (Figure 2a). This polarisation was even more pronounced amongst activated cTfh cells (Figure 2b, Supplementary figure 3h–j). Compared to day 0, the frequency of the cTfh1 cells was strongly increased by day 14 and day 28 of the immune response to YF‐17D, whereas the frequency of cTfh17 cells and also CXCR3^−^CCR6^−^ cTfh2 cells were decreased within the activated CD38^+^ compartment of circulating T follicular helper cells (Figure 2b). Similarly, the frequency of the cTfh1 cells within ICOS^+^CD38^+^, ICOS^+^PD‐1^+^ and PD‐1^+^CD38^+^ cTfh cells was strongly increased on day 14 and day 28 after vaccination with YF‐17D (Supplementary figure 3h–j). To gain further insight into the functionality of the activated cTfh cell subsets, we performed intracellular cytokine staining of peripheral blood mononuclear cells (PBMCs) that had been restimulated with PMA and ionomycin (Figure 2c). Activated cTfh cells on day 14 post‐vaccination showed high frequencies of IFNγ‐producing cTfh1 cells and lower frequencies of IFNγ‐producing cTfh1‐17 and cTfh2 cells after non‐specific re‐stimulation (Figure 2c). IL‐4^+^ cells were found in the cTfh1 and cTfh2 cell subsets while IL‐17 was produced by cTfh1‐17 and cTfh17 cells (Figure 2c). IL‐21^+^ and IL‐2^+^ cells were detected in all four subsets, although IL‐21‐ as well as IL‐2‐producing cells were particularly enriched in the cTfh1 cell population (Figure 2c).

### Kinetics of circulating CXCR5^−^ T memory cells following YF‐17D vaccination

**Figure 3:**
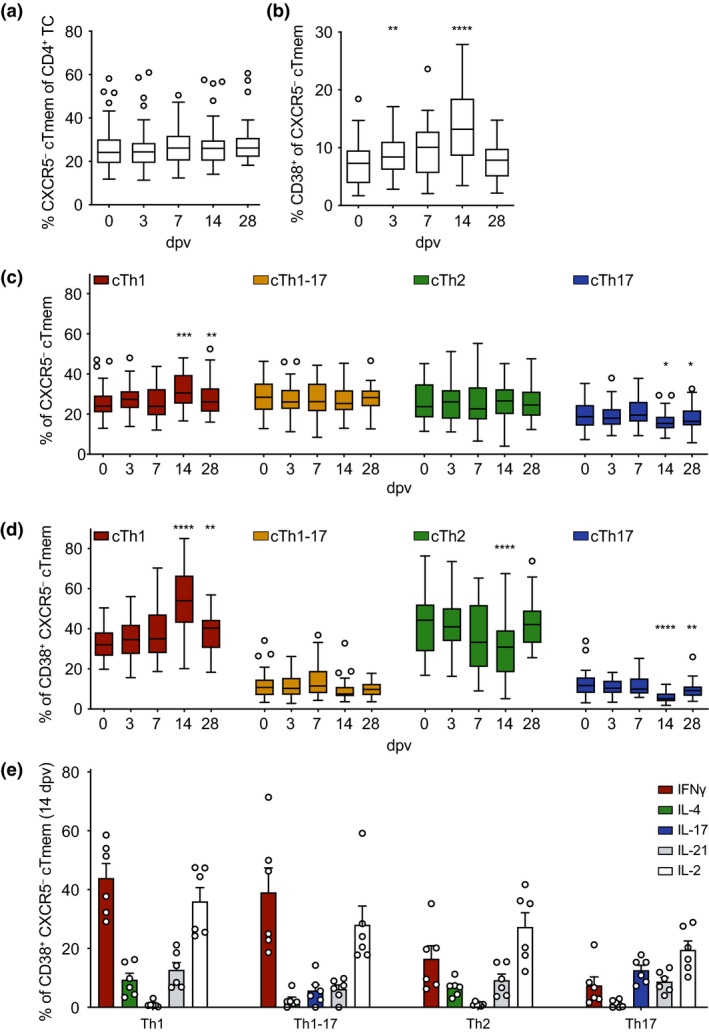
Kinetics, activation and subset composition of circulating CXCR5^−^ T memory cells after YF‐17D vaccination. PBMCs isolated before (day 0) and at the indicated time points after YF‐17D vaccination were analysed by flow cytometry (see Supplementary figure 1 for the gating strategy). Frequencies of **(a)** circulating CXCR5^−^ Tmem cells, **(b)** activated CD38^+^ CXCR5^−^ cTmem cells, **(c)** CXCR5^−^ cTmem cell subsets and **(d)** activated CD38^+^ CXCR5^−^ cTmem cell subsets determined by CXCR3 and CCR6 expression are shown. **(e)** Frequencies of cytokine‐expressing CD38^+^ CXCR5^−^ cTmem cells were measured by intracellular antibody staining after re‐stimulation *ex vivo* with PMA/ionomycin on day 14 after YF‐17D vaccination. **(a–d)** Pooled data from four independent experiments with 5 to 10 study participants each are presented as Tukey boxplots showing the median with the 25th and 75th percentile (*n* = 33) and whiskers and outliers calculated as highest and lowest observation below/above 1.5 times interquartile range. Data points below/above 1.5 times interquartile range are displayed individually. Statistical analysis was performed using RM one‐way ANOVA and Dunnett's multiple comparison analysis to compare indicated time points to day 0. *P < 0.0332, **P < 0.0021, ***P < 0.0002, ****P < 0.0001. **(e)** Representative data of one of four separately performed experiments are shown as mean and SEM with each dot representing one donor (*n* = 6).

To further analyse the kinetics of the CD4^+^ T cell response after YF vaccination, we also assessed the frequencies of circulating CXCR5^−^ cells amongst CD4^+^CD45RO^+^ memory T cells (CXCR5^−^ cTmem; gating strategy in Supplementary figure 1a). Although there were no significant changes in the absolute number of CD4^+^ T cells, nor in the frequency of memory CD4^+^ T cells, nor in the frequency of CXCR5^−^ cTmem cells amongst CD4^+^ T cells in the blood of YF vaccinees at the investigated time points after vaccination (Supplementary figure 3a, b and e, Figure 3a), the absolute number of CXCR5^−^ cTmem cells was slightly decreased on day 3 and slightly increased on day 14 and day 28 (Supplementary figure 3d). While the frequencies of total CXCR5^−^ cTmem cells did not change significantly after YF vaccination, there was a strong transient increase in the frequency of activated CD38^+^CXCR5^−^ cTmem cells (Figure 3b, Supplementary figure 3f). The frequency of CD38^+^CXCR5^−^ cTmem cells was already increased on day 3 post‐vaccination, reaching its peak by day 14 and then declining to frequencies of activated cells comparable to pre‐vaccination levels by day 28 (Figure 3b). Coinciding with the peak of activation on day 14, polarisation of the CXCR5^−^ cTmem cells was also altered (Figure 3c, Supplementary figure 3g). The frequency of circulating CXCR3^+^CCR6^−^ Th1 cells was increased on day 14 and day 28 following YF vaccination (Figure 3c). This was at the expense of CXCR3^−^CCR6^+^ Th17 cells, which were underrepresented on day 14 and day 28 compared to pre‐vaccination frequencies (Figure 3c). This tendency was also observed when CXCR5^−^ cTmem cells were pre‐gated on CD38^+^ activated CXCR5^−^ cTmem cells (Figure 3d). Activated CXCR5^−^ cTmem cells were strongly polarised towards the cTh1 subtype on day 14, and this polarisation persisted until at least day 28 (Figure 3d). Along with the highest frequency of activated cTh1 cells on day 14, CXCR3^−^CCR6^−^ Th2 cells were decreased in frequency on day 14, and cTh17 cells were decreased in frequency on day 14 and to a lesser extent on day 28 after vaccination compared to day 0 (Figure 3d). Intracellular cytokine staining after non‐specific re‐stimulation revealed that IFNγ was mostly produced by the Th1 and the CXCR3^+^CCR6^+^ Th1‐17 subset (Figure 3e). IL‐4 was produced by Th2 cells, but also by a fraction of Th1 cells. IL‐17‐producing cells were found within the Th1‐17 and Th17 subsets. While IL‐21‐ and IL‐2‐producing cells were detected amongst all four subsets, the Th1 compartment contained the highest frequency of IL‐2‐producing cells. Compared to CXCR5^+^ cTfh cells (Figure 2c), the frequencies of IL‐21^+^ and IL‐2^+^ cells were lower in the activated CXCR5^−^ cTmem cell compartment.

### Circulating Treg cell frequencies are increased early after YF‐17D vaccination

**Figure 4:**
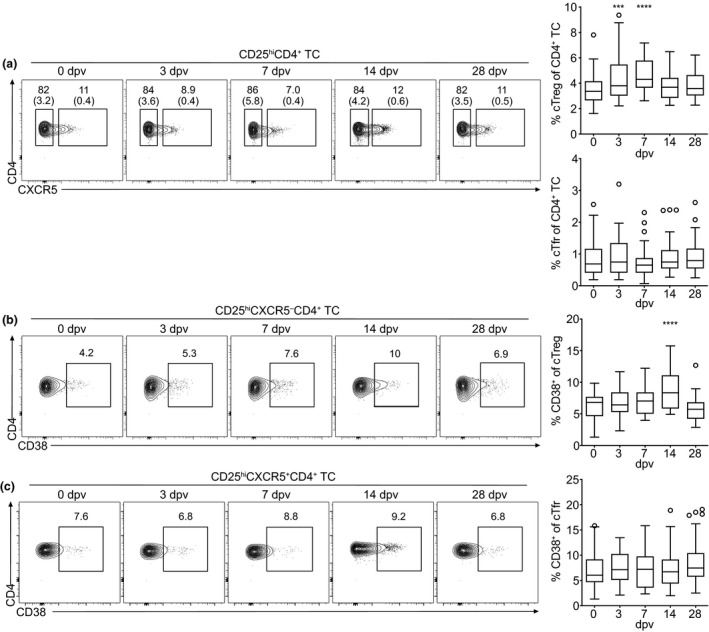
Kinetics and activation of cTreg and cTfr cells after vaccination with YF‐17D. PBMCs isolated before (day 0) and at the indicated time points after YF‐17D vaccination were analysed by flow cytometry (see Supplementary figure 1 for the gating strategy). **(a–c)** Representative contour plots and quantification of frequencies of **(a)** circulating CXCR5^−^ Treg and CXCR5^+^ Tfr cells, **(b)** activated CD38^+^ cTreg and **(c)** activated CD38^+^ CXCR5^+^ Tfr cells are shown. Gate frequencies indicate the frequency in regard to the parent gate. Gate frequencies in brackets indicate the frequency of the population in regard to the reference population as indicated above. Pooled data from four independent experiments with 5 to 10 study participants each are presented as Tukey boxplots showing the median with the 25th and 75th percentile (*n* = 33) and whiskers and outliers calculated as highest and lowest observation below/above 1.5 times interquartile range. Data points below/above 1.5 times interquartile range are displayed individually. Statistical analysis was performed using RM one‐way ANOVA and Dunnett's multiple comparison analysis to compare indicated time points to day 0. ***P < 0.0002, ****P < 0.0001.

Following the assessment of the kinetics of cTfh cells (Figures 1 and 2) and CXCR5^−^ cTmem cells (Figure 3), we next investigated circulating regulatory T cells after YF vaccination. Regulatory T cells were subdivided into CXCR5^−^ regulatory T (Treg) cells and CXCR5^+^ T follicular regulatory (Tfr) cells (see Supplementary figure 1a for gating strategy). Absolute numbers of blood CXCR5^−^ Treg cells were increased on day 14 (Supplementary figure 4a), whereas the frequency of CXCR5^−^ Treg cells amongst all CD4^+^ T cells was significantly increased early on day 3 and day 7 post‐vaccination (Figure 4a). Along with the increase in absolute numbers, the frequency of CD38^+^ activated cTreg was strongly increased on day 14 post‐vaccination but not yet on day 7 (Figure 4b). Therefore, an increase in the frequency of activated CXCR5^−^ cTmem and cTfh cells preceded the increase in the frequency of activated cTreg cells (Figures 1b, 3b and 4b). Absolute numbers of cTfr cells were slightly increased on day 28 after vaccination (Supplementary figure 4b) and frequencies did not change significantly during the immune response to YF‐17D (Figure 4a). In contrast to CXCR5^−^ Treg cells, the frequency of CD38‐expressing cTfr cells was similar at all time points investigated and did not increase in response to yellow fever vaccination (Figure 4c).

### Tracking of YFV‐specific T helper cells by MHC class II tetramer staining reveals epitope‐specific qualitative differences within cTfh and CXCR5^−^ Tmem cell subsets

**Figure 5:**
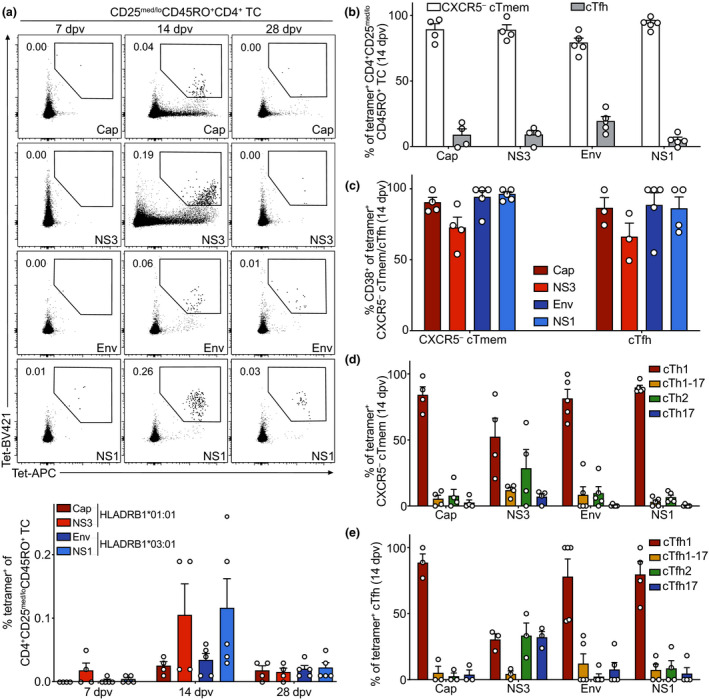
Detection and analysis of YFV‐specific CD4^+^ T cells following YF‐17D vaccination. PBMCs isolated before (day 0) and at the indicated time points after YF‐17D vaccination were analysed by flow cytometry. **(a)** Representative contour plots and quantification of frequencies of YFV‐specific CD45RO^+^ CD4^+^ T cells determined by co‐staining with BV421‐ and APC‐labelled tetramers on days 7, 14 and 28 after vaccination are shown. Tetramers were HLA‐matched (HLADRB1*01:01 or HLADRB1*03:01) and presented YFV peptides derived from the Cap or NS3, and Env or NS1, proteins, respectively. **(b–e)** Frequencies of YFV‐specific **(b)** CXCR5^−^ cTmem and cTfh cells, **(c)** CD38‐expressing activated CXCR5^−^ cTmem and cTfh cells, **(d)** CXCR5^−^ cTmem and **(e)** cTfh subsets were determined on day 14 after vaccination. Pooled data from three independent experiments are shown as mean with SEM, with each dot representing one donor (HLADRB1*01:01 *n* = 4, HLADRB1*03:01 *n* = 5). Donors with no cell counts for Tfh cells in **b** were excluded from the analysis in **c** and **e**.

We took advantage of a previously published list of YFV‐specific tetramers to directly investigate YF‐17D antigen‐specific CD4^+^ T cells *ex vivo*. Two HLA types (HLA‐DRB1*03:01 and DRB1*01:01) frequently represented in our cohort were chosen for generation of tetramers in combination with two pre‐validated YFV peptides for each HLA type. Antigen‐specific cells were determined by co‐staining with a BV421‐ and an APC‐labelled tetramer to prevent inclusion of unspecifically bound cells in the analysis. YFV‐specific CD4^+^ T cells reactive to peptides derived from the viral proteins Cap, NS3, Env and NS1 were detectable in the blood of vaccinees on day 14 after vaccination while almost no cells specific to the antigens tested could be seen on day 7 yet (Figure 5a). This is in accordance with previous publications that detected YFV‐specific cells by CD40L or tetramer staining 2 weeks after vaccination.^21^, ^26^ On day 28, the frequency of antigen‐specific cells decreased and only few of the cells could still be detected in peripheral blood (Figure 5a). Antigen‐specific CD4^+^ T cells were mostly CXCR5^−^ cTmem cells with up to 20% CXCR5^+^ cTfh cells depending on the antigen investigated (Figure 5b). The vast majority of antigen‐specific CXCR5^−^ cTmem and cTfh cells was activated and expressed CD38 on day 14 (Figure 5c). Analysis of the subset composition of antigen‐specific cells showed that Cap‐, Env‐ and NS1‐specific CD4^+^ T cells were largely of the cTh1 and cTfh1 subset, highlighting the role of these subsets in the antiviral immune response (Figure 5d and e). In contrast, NS3‐specific CXCR5^−^ cTmem cells were also polarised into cTh2 cells (Figure 5d) and NS3‐specific cTfh cells were also polarised into cTfh2 and cTfh17 subsets (Figure 5e).

### The frequency of antibody‐secreting cells in the blood is increased following YF‐17D vaccination

**Figure 6:**
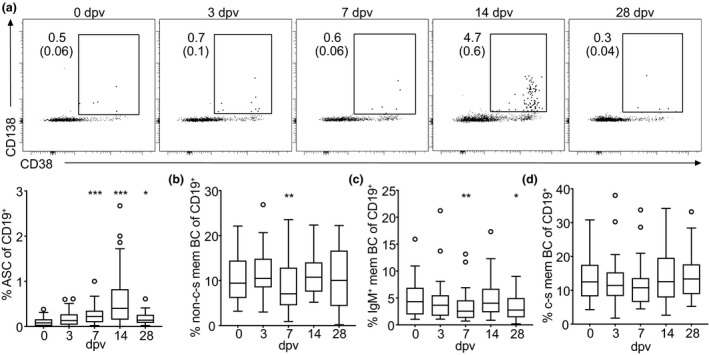
Characterisation of B cell subsets after yellow fever vaccination. PBMCs isolated before (day 0) and at the indicated time points after YF‐17D vaccination were analysed by flow cytometry (see Supplementary figure 1 for the gating strategy). **(a–d)** Quantification of frequencies and **(a)** representative contour plots of antibody‐secreting cells (ASCs), **(b)** non‐class‐switched (non‐c‐s), **(c)** IgM^+^ and **(d)** class‐switched (c‐s) memory B cell (mem BC) subsets are shown. Gate frequencies indicate the frequency in regard to the parent gate. Gate frequencies in brackets indicate the frequency of the population in regard to the reference population as indicated above. Pooled data from four independent experiments with 5 to 10 study participants each are displayed as Tukey boxplots showing the median with the 25th and 75th percentile (*n* = 33) and whiskers and outliers calculated as highest and lowest observation below/above 1.5 times interquartile range. Data points below/above 1.5 times interquartile range are displayed individually. Statistical analysis was performed using RM one‐way ANOVA and Dunnett's multiple comparison analysis to compare indicated time points to day 0. *P < 0.0332, **P < 0.0021, ***P < 0.0002.

As Tfh and B cells orchestrate the antibody response, we set out to analyse B cell subpopulations following YF vaccination. CD19^+^ cells were gated on IgD, CD27, CD38, CD138 and IgM to differentiate B cell subsets (see Supplementary figure 1c for gating strategy). Similar to CD8^+^ T cells, the total number of CD19^+^ B cells was decreased on day 3 and day 7 after yellow fever vaccination in the blood (Supplementary figure 5a). While there were no major changes observed in the frequency of CD27^+^ memory B cells, naïve B cells (IgD^+^CD27^−^CD38^−^) and transitional B cells (IgD^+^CD27^−^CD38^+^; Supplementary figure 5b–d), the frequency of antibody‐secreting cells (ASCs), which include plasmablasts and plasma cells (IgD^−^CD27^+^CD138^+^), was substantially increased on day 7 and peaked on day 14 after vaccination (Figure 6a), which confirmed previous publications.^21^ CD27^+^ memory B cells were further subdivided into memory B cell subsets. Non‐class‐switched memory B cells (IgD^+^CD27^+^) were decreased in their frequency amongst CD19^+^ cells on day 7 (Figure 6b), whereas IgM^+^ memory B cells (IgD^−^IgM^+^CD27^+^CD138^−^) were decreased in their frequency on day 7 and day 28 (Figure 6c). The frequency of class‐switched memory B cells (IgD^−^IgM^−^CD27^+^CD138^−^) amongst CD19^+^ cells did not change significantly after vaccination with YF‐17D compared to pre‐vaccination levels (Figure 6d).

### Distinct subsets of cTfh cells correlate with neutralising antibody titres

**Figure 7:**
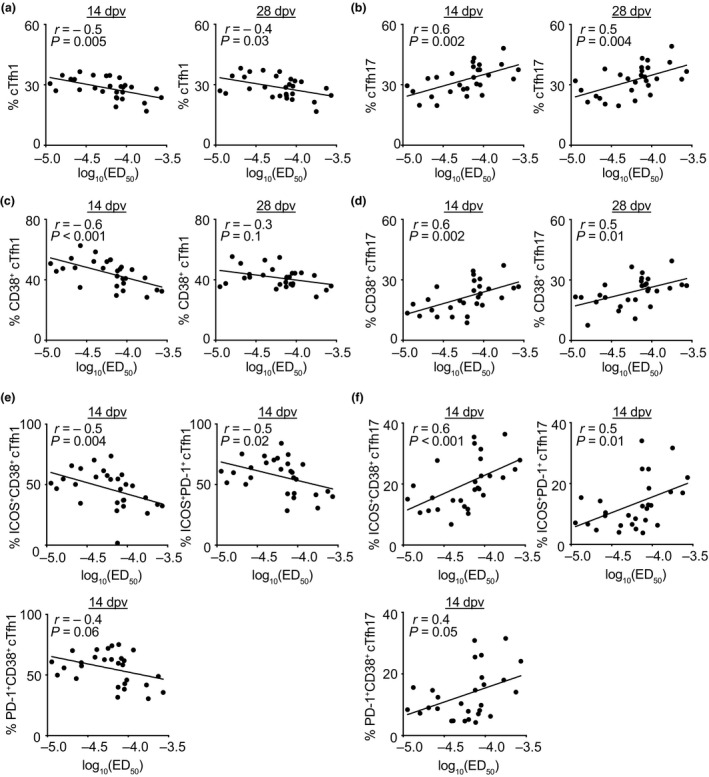
Neutralising antibody titres correlate with Tfh cell subset frequencies after yellow fever vaccination. **(a–f)** The level of neutralising antibodies at day 28 after YF‐17D vaccination was measured by focus reduction neutralisation tests and is shown as log_10_(ED_50_) representing the dilution of serum called effective dose (ED) leading to 50% reduction of foci in the test. The ED50 was correlated with the frequencies of **(a)** Tfh1, **(b)** Tfh17, **(c)** activated CD38^+^ Tfh1 and **(d)** activated CD38^+^ Tfh17 cells on days 14 and 28 after vaccination, as well as **(e)** cTfh1 cells and **(f)** cTfh17 cells expressing different combinations of the activation markers ICOS, CD38 and PD‐1 on day 14 post‐vaccination as determined by flow cytometry. Spearman's rank correlation coefficients were used to calculate correlations, and *r*‐ and *P*‐values are indicated. Linear regressions are shown as lines and each dot represents one donor (*n* = 28).

Long‐lasting immunity against YF virus following vaccination requires both cellular and humoral immunity. Even though more than 95% of vaccinated people develop long‐lasting protective neutralising antibody titres as early as 10 days after vaccination, the titres amongst individuals vary.^7^, ^21^ In order to determine prognostic markers for the titre of neutralising antibodies present on day 28 after vaccination, we correlated the neutralising antibody titre with several Tfh cell populations on day 3, day 7, day 14 and day 28 after vaccination. The titre was determined as the dilution of sera which showed a 50% reduction of the focus count in a focus reduction neutralisation test (FRNT). While none of the frequencies of CXCR5^−^ cTmem cells as well as none of the pre‐vaccination (0 dpv) frequencies of cTfh cell parameters correlated with the neutralising antibody titre (data not shown), we found that the frequencies of cTfh1 and cTfh17 cells correlated with the neutralising activity measured in the serum of vaccines (Figure 7a and b). cTfh1 cell frequencies on day 14 and day 28 correlated positively with the measured neutralising activity [higher effective dose (ED) of neutralising antibodies in the serum; Figure 7a]. This meant that in patients with higher frequencies of cTfh1 cells sera had to be diluted more, in order to decrease neutralising activity to 50% (ED_50_) and therefore contained higher levels of neutralising antibody in the serum. In contrast, cTfh17 cell frequencies on day 14 and day 28 were negatively correlated with the measured neutralising activity (Figure 7b). These findings could also be confirmed by correlations of activated CD38^+^, ICOS^+^CD38^+^, ICOS^+^PD‐1^+^ and PD‐1^+^CD38^+^ cTfh1 and cTfh17 cells with the level of neutralising activity (Figure 7c–f).

## Discussion

In this study, we provided detailed analyses of lymphocyte dynamics after yellow fever vaccination, with a focus on Tfh cells. Tfh cells have to our knowledge not been investigated in this setting before. We showed that activation and polarisation of CXCR5^+^ Tfh cells and CXCR5^−^ memory T helper cells change in response to vaccination with the live‐attenuated yellow fever vaccine YF‐17D and that the frequencies of cTfh1‐polarised cells is predictive for the level of neutralising activity in the serum of vaccinees. Alterations in cTfh and cTmem frequencies followed similar patterns, mirrored the changes seen in regulatory T cells and CD8^+^ T cells after YF vaccination and were accompanied by an increase in the frequency of ASCs. The peak of activation was detected on day 14 for most lymphocyte subsets investigated. These kinetics are in accordance with previous publications that used various activation markers and their combinations, including CD38, Ki67 and HLA‐DR^18^, ^20^, ^21^, ^27^ or TCR repertoire sequencing, to track the YF‐17D response of CD8^+^ and CD4^+^ T cells.^28^, ^29^

In our study, the activation peak at day 14 post‐vaccination was preceded by a drop in the absolute numbers of CXCR5^−^ cTmem, cTfh, CD8^+^ T cells and B cells on day 3 and day 7, which most likely reflects their recruitment to secondary lymphoid organs and has also been observed before.^21^ The drop in the number of CXCR5^−^ cTmem and cTfh cells leads to an increased proportion of cTreg cells amongst CD4^+^ T cells on day 3 and day 7 post‐vaccination, but in addition to this passive shift early on, the frequency of activated cTreg cells was increased 2 weeks after vaccination as described previously.^27^ Interestingly, an increase in the frequency of activated CD38‐expressing CXCR5^−^ cTmem, cTfh and CD8^+^ T cells was evident on day 7 post‐vaccination and persisted until day 28 for cTfh and CD8^+^ T cells. In contrast, Treg cells, which inhibit and control ongoing immune reactions, were activated and expanded in the blood with a delay compared to activated CD4^+^ and CD8^+^ T cells.

Along with the activation of CD4^+^ T cells upon YF vaccination, we found a strong polarisation of CXCR5^−^ cTmem and cTfh cells and especially activated CXCR5^−^ cTmem and cTfh cells towards CXCR3‐expressing, IFNγ‐secreting Th1 and Tfh1 cells. Interestingly, this Th1 and Tfh1 polarisation was at the expense of cTh17 and cTfh17 cells. As Th1 cells are the primary CD4^+^ T cell subset involved in antiviral responses^30^, ^31^ the dominance of the cTfh1 subtype is not unexpected and had been described previously in infections with HIV and HCV and after influenza vaccination.^25^, ^32^, ^33^, ^34^, ^35^, ^36^ In line with these studies, we found a positive correlation of the cTfh1 subset size with the neutralising activity in the blood and an inverse correlation with the cTfh17 subset. This strengthens our interpretation that (c)Tfh1 cells are the major B cell helper cell type in the anti YF‐17D immune response.

Although cTfh1 cells were originally described as inferior B cell helpers in terms of antibody production *ex vivo* when compared to cTfh2 and cTfh17 cells,^11^ the type of infection and the selective subclass and specificity required for protective antibodies may eventually determine which cTfh cell subtype is relevant and prognostic for the outcome of an infection or the vaccination success, respectively. This could also explain why a high frequency of ASCs does not necessarily correlate with a high titre of neutralising antibodies.

Tfh1 cells might not only influence the outcome of the humoral immune response. It has been shown in mouse studies that CD4^+^ T cells in addition to their B cell helper capabilities are also essential for conferring efficient and long‐lasting protection against wildtype YFV infection.^4^, ^37^ Tfh1 cells have been implicated in the formation of CD4^+^ T cell memory cells^9^ as well as contributing effector functions which mostly rely on cytokine expression.^38^

Cytokine expression profiles of CXCR5^−^ cTmem cells at the peak of the immune response to YF‐17D revealed that the highest fraction of IFNγ‐producing cells was found in the Th1 cell subset. Generally, we were able to confirm that activated CXCR5^−^ cTmem cell subsets expressed their subset‐determining cytokines on day 14 after yellow fever vaccination. This was also confirmed for cTfh subpopulations defined by the expression of the chemokine receptors CXCR3 and CCR6. IFNγ‐producing cells were mostly found in the cTfh1 cell subset and to a lesser extent in the cTfh1‐17 cell subset, whereas IL‐17‐producing cells were mostly found amongst cTfh17 and cTfh1‐17 cells. IL‐2 is secreted upon antigen stimulation and thereby induces proliferation.^39^ In our study, cTh1 and cTfh1 cells had the highest fraction of IL‐2 expressing cells. Interestingly, cTfh cells generally showed higher fractions of IL‐2‐expressing cells than CXCR5^−^ cTmem cells, which is in line with a previous report.^11^

Compared to activated cTfh cell subsets, CXCR5^−^ cTmem cells expressed slightly lower levels of IL‐21. It has been suggested, that cTfh1 cells lack B cell helper abilities as they only secrete very low levels of IL‐21.^25^ We could show that cTfh1 cells in response to YF vaccination secrete more IL‐21 than cTfh2 and cTfh17 cell subsets, which have been described as efficient B cell helpers *in vitro*.^11^ Possible explanations for this finding are that IL‐21 secretion by cTfh1 is induced by YF‐17D or that resting cTfh1 cells were previously used for assaying B cell helper abilities.^15^ Nevertheless, we saw a strong increase in activation markers on cTfh1 and IL‐21 might only be produced upon activation by this subset. This further argues that cTfh1 cells *in vivo* indeed have the potential and relevant cytokine production to foster the generation of neutralising antibodies after YF vaccination.

The polarisation and activation kinetics of circulating Tfh and CXCR5^−^ Tmem cells after YF vaccination was further confirmed in YF‐17D‐specific CD4^+^ T cells defined by MHC II tetramer staining. Tetramer‐binding CXCR5^−^ cTmem and cTfh cells were detectable on day 14 post‐vaccination and expressed the activation marker CD38, validating that indeed, a high fraction of T cells, which expressed the CD38‐marker upon vaccination, are specific for the YF virus. Furthermore, tetramer‐positive CD4^+^ T cells were mostly of the Th1 and Tfh1 cell subtype, again highlighting the role of those subsets in the immune response to YF‐17D. cTfh cells thereby recognised the same peptide epitopes of the capsid and envelop protein, as well as non‐structural protein 1 that had been described to dominate the CXCR5^−^ Th1 cell subset.^26^ CD4^+^ T cells recognising the NS3 49–65 peptide epitope did not show the clear Th1/Tfh1 polarisation dominance seen in cells specific for the other three antigens tested and comprised almost equal numbers of Th1/Tfh1, Th2/Tfh2 and Th17/Tfh17 cells. This argues for an epitope‐dependent component like TCR‐signalling strength influencing the cTfh differentiation into their subpopulations. The predominance of the cTfh1 differentiation by at least the most important YF‐17D antigens for neutralisation is reflected in the positive correlation of the cTfh1 frequency with the level of neutralising activity in the humoral YF‐17D‐specific immune response.

In summary, the immune response to YF‐17D is Th1‐ and Tfh1‐driven and the peak of activated CD4^+^ T cells, CD8^+^ T cells and ASCs can be detected 2 weeks after vaccination in the blood. Our data provide a predictive connection between the frequencies and polarisation of cTfh cells and the humoral immune response to live‐attenuated viral vaccines. These findings will aid the understanding of T cell and B cell responses to viral infections and might help to implement the induction of a potent cTfh1 response in vaccine development processes.

## Methods

### Study participants and vaccination

The study protocol was approved by the Institutional Review Board of the Medical Faculty of LMU Munich and adhered to the most recent version of the declaration of Helsinki. Participants were not previously immunised against YF and had not been naturally exposed to the wild type YFV. After giving informed consent, 34 individual healthy donors (Supplementary table 1) aged 18–38 received a single vaccine shot of the live‐attenuated 17D yellow fever virus strain (0.5 mL of Stamaril; Sanofi Pasteur, Lyon, France) subcutaneously at the Division of Infectious Diseases & Tropical Medicine at LMU Munich. Blood samples were collected directly prior to vaccination and on days 3, 7, 14 and 28 after vaccination.

### Human cell preparation

Absolute blood cell numbers in patient samples were determined on a Sysmex XN 1000/9000 clinical diagnostics system according to the manufacturer's recommendations. PBMCs were isolated from heparinised human whole blood by Ficoll‐Hypaque (Biochrom, Berlin, Germany) gradient in SepMate tubes (STEMCELL, Vancouver, Canada) within 6 h from blood draw. Cells were further analysed directly after isolation. Sera were stored at −80°C until further use.

### Phenotypic analyses of lymphocytes

Peripheral blood mononuclear cells were analysed by flow cytometry as previously described.^40^ In Brief, PBMCs were first incubated with Fc receptor blocking agent (Miltenyi, Bergisch Gladbach, Germany) and then stained at 4°C with a T cell and a B cell panel (antibodies used for the panels with clone and manufacturer information can be found in Supplementary table 2). Dead cells were excluded with the fixable viability dye eFluor 780 (eBioscience/Thermo Fisher, Waltham, MA, USA). PBMCs were washed and then fixed with 1% paraformaldehyde prior to acquisition. Events were recorded on a BD LSRFortessa (Becton Dickinson, Franklin Lakes, NJ, USA), and data were analysed with FlowJo software version 10 (FlowJo LLC, Ashland, OR, USA).

### Stimulation of *ex vivo* isolated lymphocytes

In all, 3–5 million PBMCs were stained with antibodies against CD4, CXCR5, CXCR3 and CCR6 (for clones and manufacturer, see Supplementary table 2 ‘cytokine panel’) after blocking of the Fc receptors and before stimulation with PMA/ionomycin to reduce loss of chemokine receptor signals during the stimulation. After washing the cells were stimulated with 50 nmol L^−1^ phorbol 12‐myristate 13‐acetate (PMA) and 1 µmol L^−1^ ionomycin (both Sigma‐Aldrich, St. Louis, MO, USA) at 37°C in complete RPMI (cRPMI; 10% foetal calf serum, 10 mm HEPES, 2 mm L‐glutamine, 1 mm sodium pyruvate, 1× non‐essential amino acids, 50 µm β‐mercaptoethanol, 100 U penicillin–streptomycin). After 2.5 h, 5 µg mL^−1^ brefeldin A (Sigma‐Aldrich) was added for additional 2.5 h. The stimulated cells were first incubated with antibodies against CD45RO CD3, CD19, CD8a, CD56, CD14 and CD38 (for clones and manufacturer, see Supplementary table 2 ‘cytokine panel’). Dead cells were excluded with the fixable viability dye eFluor 780 (eBioscience/Thermo Fisher). After fixation and permeabilisation with the Fixation/Permeabilization Solution Kit (‘Cytofix/Cytoperm’; Becton Dickinson) according to the manufacturer's protocol, the cells were stained intracellularly with antibodies against different cytokines (see Supplementary table 2 ‘cytokine panel’).

### HLA typing

Human leukocyte antigen (HLA) typing was performed using DNA isolated from buffy coats obtained directly prior to vaccination and either Sanger or next‐generation sequencing (NGS). Typing results were reported in a 2‐field resolution (Sanger on 3130xl Genetic Analyzer; Applied Biosystems, Foster City, CA, USA) or a 3‐field resolution (NGS Ion Chef System and Ion personal genome machine, Life Technologies/Thermo Fisher, Waltham, MA, USA). Sanger sequencing was realised by a home‐made PCR amplification strategy of class II (exons 2–4). Sequence raw data were processed either by uType (Thermo Fisher, Waltham, MA, USA) software (Sanger) or by NGSengine (NGS; GenDx, Utrecht, the Netherlands) for HLA type creation.

### Determination of *ex vivo* frequency of YFV‐specific CD4^+^ T cells using MHC multimer staining

In all, 3–10 million PBMCs were incubated with 20 µg mL^−1^ APC‐ and 20 µg mL^−1^ BV421‐labelled MHC class II tetramers (NIH Tetramer Core Facility, Atlanta, GA, USA) in 100 µL cRPMI at RT simultaneously. The peptides (JPT Peptide Technologies, Berlin, Germany) used for HLA‐DRB1 tetramer generation have been published^26^: Cap 49‐65 (FFFLFNILTGKKITAHL, HLADRB1*01:01 tetramer), NS3 49‐65 (HTMWHVTRGAFLVRNGK, HLADRB1*01:01 tetramer), Env 43‐59 (ISLETVAIDRPAEVRKV, HLADRB1*03:01 tetramer) and NS1 85‐101 (DISVVVQDPKNVYQRGT, HLADRB1*03:01 tetramer). Cells were washed and then stained with the antibodies listed in Supplementary table 2 ‘tetramer panel’. Dead cells were excluded with the fixable viability dye eFluor 780 (eBioscience/Thermo Fisher). PBMCs were washed and then fixed with 1% paraformaldehyde prior to acquisition. Events were recorded on a BD LSRFortessa, and data were analysed with FlowJo software version 10.

### Determination of YF‐17D‐neutralising antibodies

For determination of neutralising antibody titre, blood was collected in serum collection tubes (S‐Monovette Z‐Gel; Sarstedt, Nuembrecht, Germany), centrifuged and frozen at −80°C until further use. The neutralising antibody titre was determined by the focus reduction neutralisation test (FRNT) as previously described.^41^ Briefly, about 100 focus‐forming units from a YF‐17D virus stock were incubated with equal amounts of inactivated and serially diluted donor sera for 1 h at 37°C. The mixture was then added to Vero cells (25 000 cells per well in a 96‐well plate) and foci were left to develop under a layer of 1.5% methylcellulose (Sigma M0512, 4000 cP viscosity; Sigma‐Aldrich) for 2 days at 37°C. After fixation of the cells with 5% formaldehyde and blocking with 50 mM NH_4_Cl, the foci were stained with the Flavivirus anti‐E 4G2 antibody (CG 0042; Clonegene, Atlanta, GA, USA) and an anti‐mouse horseradish peroxidase (HRP)‐conjugated secondary antibody (7076S; Cell Signaling, Danvers, MA, USA). The foci were developed with 3,3′‐diaminobenzidine (DAB; D5905; Sigma‐Aldrich) and counted using the EliSpot Reader ELR04 SR (AID Autoimmun Diagnostika GmbH, Strassberg, Germany). To control for unspecific serum effects, we took advantage of our longitudinal study design and used the serum taken directly prior to vaccination for each donor as a donor‐specific negative control that was diluted in the same way as the tested serum samples from day 28. The number of foci present in the pre‐vaccination serum was then set as 100% and the % of reduction was calculated for each dilution step as 1 − (number of foci in serum from day 28/number of foci in the pre‐vaccination serum). Neutralisation curves were fitted by nonlinear regression analysis using Prism 8 (GraphPad, La Jolla, CA, USA) software and 50% FRNT values (designated ED_50_) values were interpolated from the curves. 28 of the 32 titres were clustered over a 10‐fold ED_50_ range, but the two highest and two lowest ED_50_ data points were more than threefold higher or lower than this range and were thus classified as outliers and excluded from the correlation analysis shown in Figure 7.

### Statistics

Appropriate statistical analyses were performed with Prism 8 software (GraphPad) and are specified in each corresponding figure caption.

## Conflict of interest

The authors declare no conflict of interest.

## Author contributions

JEH designed, performed and analysed all experiments, interpreted the data and wrote the manuscript together with JA; JA, MKS, MZ, LL and KW performed experiments and analysed data; ABK and SR initiated the yellow fever vaccination cohort and contributed to the interpretation of data, and SR co‐wrote the manuscript; JA, ABK, MP, MH, FvS, AD and SR provided clinical samples, and JA, HK and GB‐S contributed to the interpretation of data; MP served as clinical study investigator and performed vaccinations; DB conceived the project, interpreted the data, wrote the manuscript and provided overall direction of the study; and all authors critically reviewed the manuscript and approved the final version.

## Supporting information

Supplementary figures 1‐5
Supplementary tables 1 and 2
