# Supplementary material for: Dynamic changes in circulating T follicular helper cell composition predict neutralising antibody responses after yellow fever vaccination
Source: Clin Transl Immunology. 2020 May 13;9(5):e1129. doi: 10.1002/cti2.1129 (PMC7221214; doi:10.1002/cti2.1129)
Supplement: Supplementary file 1 — Supplementary figures 1‐5 Supplementary tables 1 and 2 [file CTI2-9-e1129-s001.pdf]

Supporting Information

Dynamic changes in circulating T follicular helper cell composition predict neutralizing antibody responses after yellow fever vaccination

Johanna E. Huber, Julia Ahlfeld, Magdalena K. Scheck, Magdalena Zaucha, Klaus Witter, Lisa Lehmann, Hadi Karimzadeh, Michael Pritsch, Michael Hoelscher, Frank von Sonnenburg, Andrea Dick, Giovanna Barba-Spaeth, Anne B. Krug, Simon Rothenfußer, Dirk Baumjohann

Supplementary figure 1

Supplementary figure 2

Supplementary figure 3

Supplementary figure 4

Supplementary figure 5

Supplementary table 1

Supplementary table 2

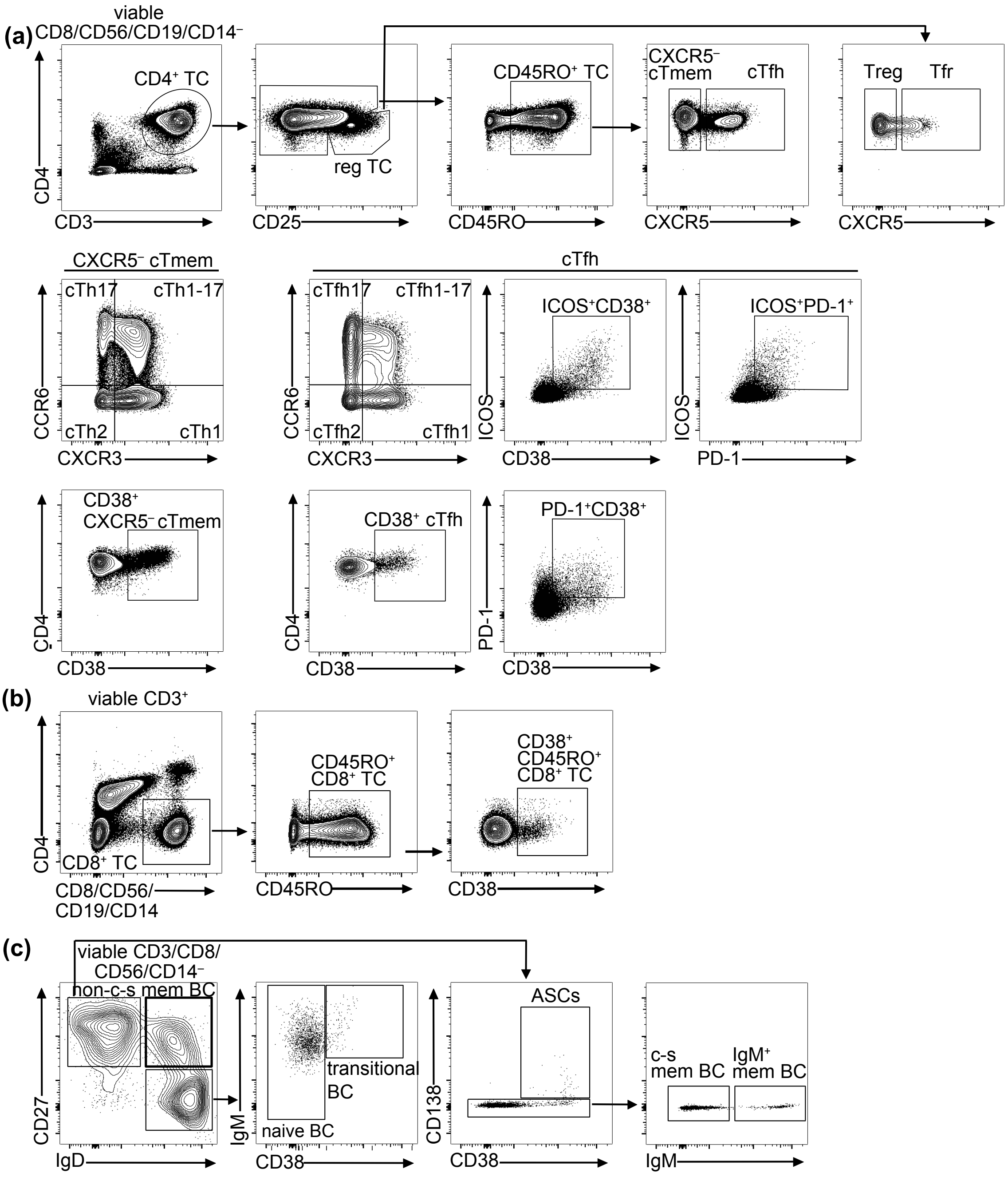

Supplementary figure 1. Gating strategy of T and B cells from human PBMCs. (**a-c**) PBMCs were isolated from whole blood, stained with mAbs and a viability dye to exclude dead cells and then analyzed by flow cytometry. (a) CD14-, CD56-, CD19-, and CD8-expressing cells were excluded to gate on CD3^{+}CD4^{+} cells. CD4^{+} T cells (TC) were then gated according to CD25 and CD4 expression and CD25^{hi} cells were defined as regulatory T cells. An average of 92% of those cells did not express CD127 (data not shown). Regulatory T cells were separated into CXCR5^{-} regulatory T (Treg) cells and CXCR5^{+} T follicular regulatory cells (Tfr). CD4^{+} T cells expressing low and intermediate levels of CD25 were gated on the memory marker CD45RO and then on CXCR5 to discriminate between CXCR5^{-} circulating T memory cells (CXCR5^{-} cTmem) and CXCR5^{+} circulating T follicular helper cells (cTfh). Those two memory subsets can be further subdivided into Th1, Th2, Th1-17, and Th17 or Tfh1, Tfh2, Tfh1-17, and Tfh17 according to their CXCR3 and CCR6 expression expression (Morita et al., 2011) or analyzed according to their activation status with CD38, ICOS, and PD-1. (b) CD8^{+} T cells were gated as CD8/CD19/CD56/CD14^{+}CD3^{+}CD4^{-} cells and their activation was assessed by gating on CD38 on CD45RO^{+} memory CD8^{+} T cells. (c) CD3-, CD14-, CD56-, and CD8-expressing cells were excluded to gate on CD19^{+} cells. B cells (BC) were then gated on IgD and the memory marker CD27, with IgD^{+}CD27^{+} B cells defined as non-class-switched memory (non-c-s mem) B cells. IgD^{+}CD27^{-} B cells were divided into CD38-expressing transitional B cells and CD38^{-} naïve B cells while IgD^{-}CD27^{+} B cells were gated on CD38^{+}CD138^{+} antibody-secreting cells (ASCs) comprised of plasma cells and plasmablasts, IgM^{+} memory B cells and IgM^{-} class-switched memory (c-s mem) B cells.

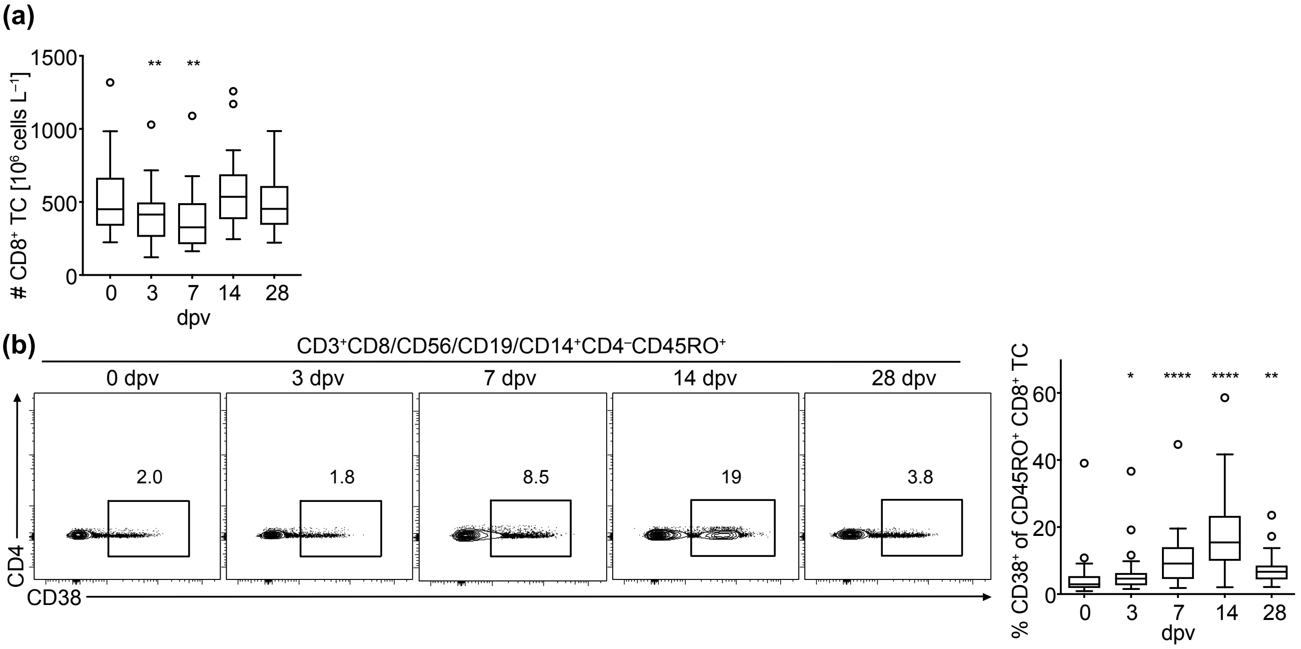

Supplementary figure 2. Kinetics of CD8+ T cells after YF-17 vaccination. (**a**) Absolute numbers of CD8+ T cells after yellow fever vaccination. (**b**) Representative contour plots and quantification of frequencies of CD38-expressing activated memory CD8+ T cells on indicated days following vaccination with YF-17D. Pooled data from three (a) or four (b) independent experiments are displayed as Tukey boxplots showing the median with 25th and 75th percentile (a n = 27 and b n = 33 donors). and whiskers and outliers calculated as highest and lowest observation below/above 1.5 times interquartile range. Data points below/above 1.5 times interquartile range are displayed individually. Statistical analysis was performed using repeated-measure (RM) one-way ANOVA and Dunett's multiple comparison analysis to compare indicated time points to d0. **P* < 0.0332, ***P* < 0.0021, *****P* < 0.0001

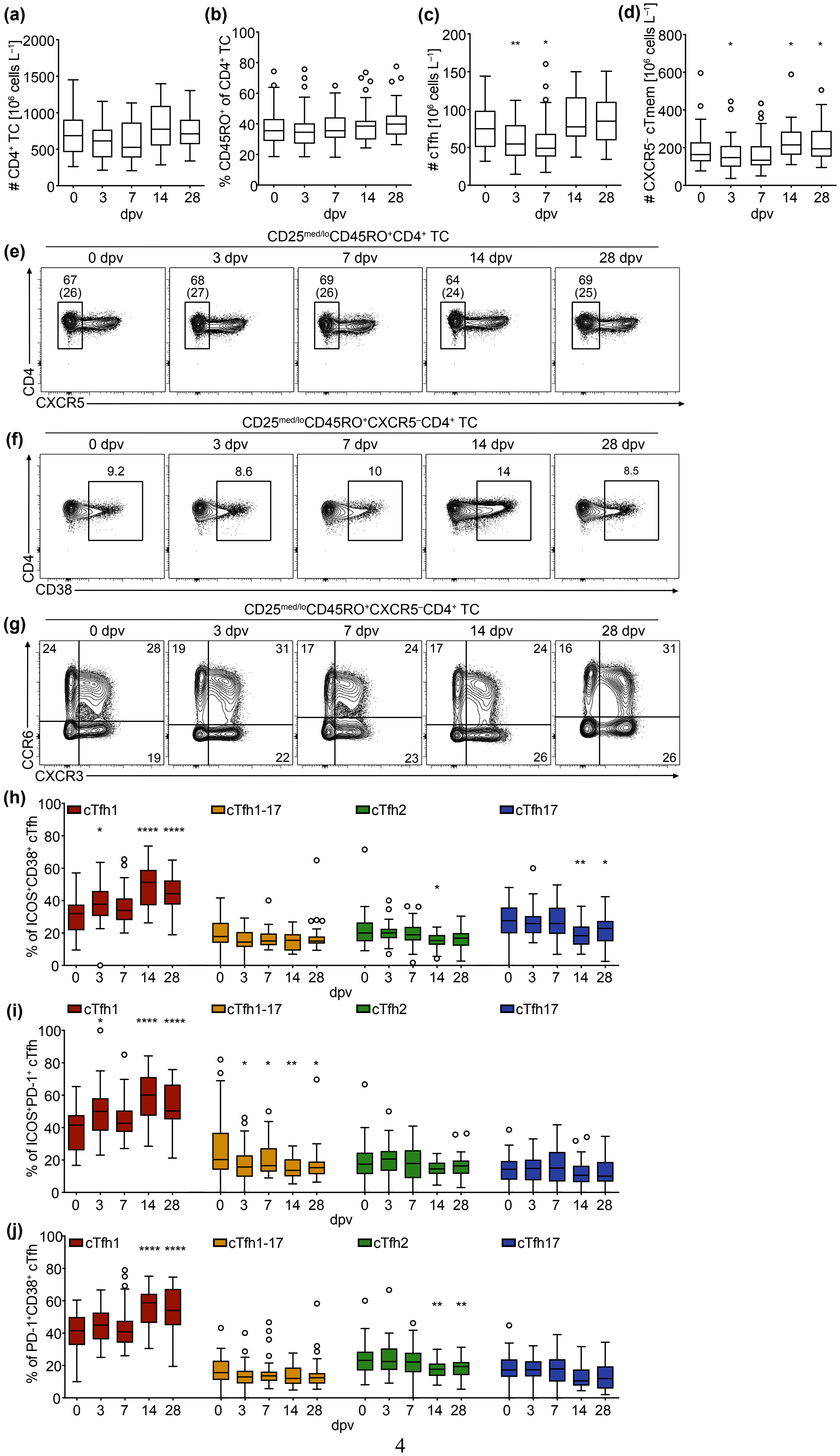

(a)
CD4+ TC [10^6 cells L^-1]
dpv
(b)
% CD45RO+ of CD4+ TC
(c)
cTfh [10^6 cells L^-1]
(d)
CXCR5- cTmem [10^6 cells L^-1]
CD25med/loCD45RO+CD4+ TC
(e)
0 dpv
3 dpv
7 dpv
14 dpv
28 dpv
67 (26)
68 (27)
69 (26)
64 (24)
69 (25)
CD4
CXCR5
CD25med/loCD45RO+CXCR5-CD4+ TC
(f)
9.2
8.6
10
14
8.5
CD38
(g)
CCR6
CXCR3
(h)
cTfh1
cTfh1-17
cTfh2
cTfh17
% of ICOS+CD38+ cTfh
(i)
% of ICOS+PD-1+ cTfh
(j)
% of PD-1+CD38+ cTfh

Supplementary figure 3. The CD4$^+$ T cell response after yellow fever vaccination. (**a**) Absolute numbers of CD4$^+$ T cells, (**b**) quantification of frequencies of CD45RO$^+$ memory CD4$^+$ T cells, (**c**) and absolute numbers of cTfh and (**d**) CXCR5$^-$ cTmem cells on indicated days after yellow fever vaccination. (**e-g**) Representative contour plots of the frequencies of (e) CXCR5$^-$ cTmem cells amongst CD4$^+$ T cells, (f) CD38-expressing activated CXCR5$^-$ cTmem cells, and (g) subsets of CXCR5$^-$ cTmem cells determined by CXCR3 and CCR6 staining corresponding to Figure 3. (**h**-**j**) Quantification of frequencies of (h) ICOS$^+$CD38$^+$, (i) ICOS$^+$PD-1$^+$, and (j) PD-1$^+$CD38$^+$ cTfh cell subsets . Pooled data from three (a, c, d) or four (b, h, i, j) independent experiments are displayed as Tukey boxplots showing the median with 25th and 75th percentile (a, c, d n = 27 and b, h, i, j n = 33 donors). Statistical analysis was performed using RM one-way ANOVA and Dunett's multiple comparison analysis to compare indicated time points to d0. **P* < 0.0332, ***P* < 0.0021, *****P* < 0.0001

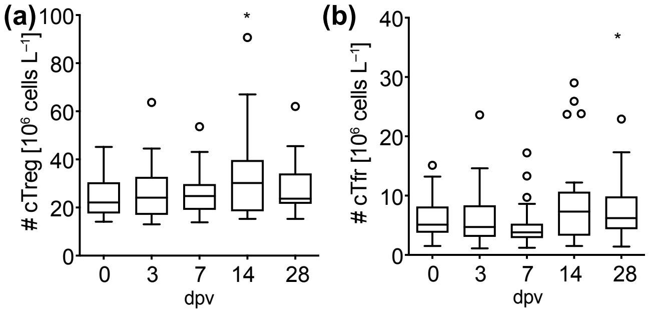

Supplementary figure 4. Circulating regulatory CD4^{+} T cells after vaccination with YFV-17D. (**a**, **b**) Absolute numbers of (a) CXCR5^{-} Treg cells and (b) CXCR5^{+} Tfr cells in the blood of yellow fever vacinees at indicated time points. Pooled data from three independent experiments are displayed as Tukey boxplots showing the median with 25th and 75th percentile (n = 27 donors). Statistical analysis was performed using RM one-way ANOVA and Dunett's multiple comparison analysis to compare indicated time points to d0. *$P < 0.0332$.

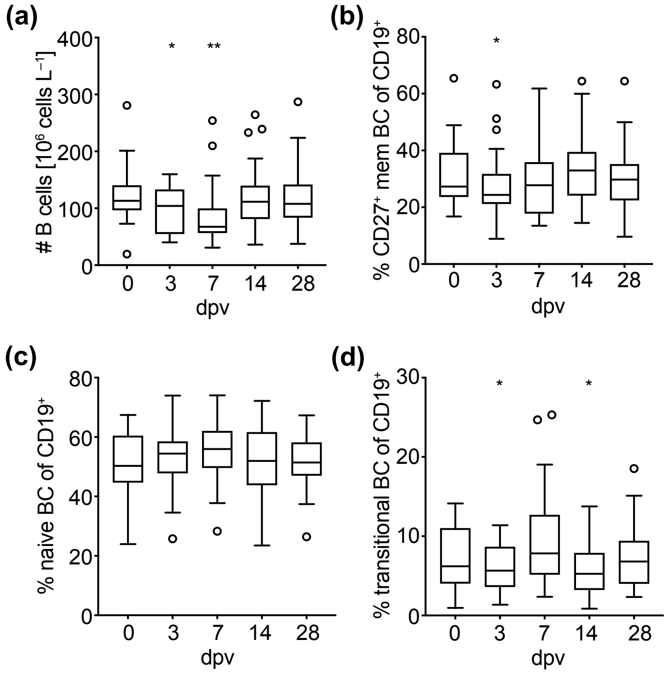

Supplementary figure 5. B cells and B cell subsets after vaccination with YFV-17D. (**a**) Absolute numbers of B cells in the blood of yellow fever vacinees at indicated time points. (**b-d**) Quantification of frequencies of (b) CD27-expressing memory B cells, (c) naïve B cells, and (d) transitional B cells after yellow fever vaccination. Pooled data from three (a) or four (b, c, d) independent experiments are displayed as Tukey boxplots showing the median with 25th and 75th percentile (a n = 27 and b, c, d n = 33 donors). Statistical analysis was performed using RM one-way ANOVA and Dunett's multiple comparison analysis to compare indicated time points to d0. *$P < 0.0332$, **$P < 0.0021$.

Study participant	Gender	Age at vaccination (years)	HLA-Type (MHC class II)					
			DRB1*-1	DRB1*-2	DQB1*-1	DQB1*-2	DPB1*-1	DPB1*-2
1	f	23	01:01	14:54	05:01	05:03	02:01	04:02
2	m	24	11:01		03:01	03:02	03:01	04:01
3	f	29	10:01	15:01	05:01	06:02	03:01	04:01
4	f	23	04:01	15:01	03:01	06:02	04:01	
5	m	27	03:01	04:01	02:01	03:01	04:01	
6	f	30	04:02	07:01	02:02	03:02	01:01	04:01
7	f	23	07:01		02:02		02:01	04:01
8	f	30	07:01	15:01	02:02	06:02	04:01	
9	m	41	07:01		02:02	03:03	01:01	04:01
10	m	33	12:01	13:01	03:01	06:03	04:02	05:01
11	f	21	13:01	15:01	06:02	06:03	04:01	11:01
12	f	22	03:01	09:01	02:01	03:03	01:01	
13	m	22	08:01	15:02	04:02	06:01	03:01	04:02
14	m	20	01:01	04:01	03:01	05:01	04:01	
15	f	23	03:01:01	11:04:01	02:01:01	03:01:01	01:01:01	04:01:01
16	f	41	01:01:01	03:04:01	02:01	05:01	02:01:02	04:02:01
17	m	21	01:01:01	03:01:01	02:01:01	05:01:01	10:01:01	13:01:01
18	f	37	01:01	08:01	04:02	05:01	04:01	
19	f	23	03:01	11:01	02:01	03:01	02:01	04:01
20	f	26	11:04	13:01	03:01	06:03	02:01	20:01
21	f	30	01:01	15:02	05:01	06:01	02:01	104:01
22	f	26	03:01	12:01	02:01	03:01	04:01	17:01
23	f	26	04:01	07:01	02:02	03:01	03:01	04:01
24	m	24	03:01	13:02	02:01	06:04	03:01	20:01
25	m	44	13:01	15:01	06:02	06:03	04:01	06:01
26	f	24	03:01	14:01	02:01	05:03	01:01	04:01
27	f	31	11:04	13:01	03:01	06:03	04:02	
28	f	24	04:02	15:01	03:02	06:02	04:01	
29	f	27	11:01	16:01	03:01	05:02	04:01	04:02
30	m	23	01:01	15:01	05:01	06:02	03:01	04:01
31	f	24	04:01		03:02	04:02	02:01	04:01
32	f	23	07:01	15:02	02:02	06:01	04:01	17:01
33	f	26	08:02	12:01	03:02		05:01	
34	f	21	01:01:01	03:01:01	02:01:01	05:01:01	03:01:01	16:01:01

Supplementary table 1. Characteristics of study participants.

	Specificity	Fluorochrome	Clone	Manufacturer
T cell panel	CD4	FITC	RPA-T4	BioLegend, San Diego, USA
	CD3	PE-Cy7	OKT3	BioLegend, San Diego, USA
	CD19	PerCP-Cy5.5	SJ25C1	BioLegend, San Diego, USA
	CD8a	PerCP-Cy5.5	RPA-T8	BioLegend, San Diego, USA
	CD56	PerCP-Cy5.5	HCD56	BioLegend, San Diego, USA
	CD14	PerCP-Cy5.5	HCD14	BioLegend, San Diego, USA
	CXCR5	APC	J252D4	BioLegend, San Diego, USA
	CXCR3	BV605	G025H7	BioLegend, San Diego, USA
	ICOS	BV510	C398.4A	BioLegend, San Diego, USA
	PD-1	BV785	EH12.2H7	BioLegend, San Diego, USA
	CD25	PE/Dazzle 594	M-A251	BioLegend, San Diego, USA
	CD38	AF700	HIT2	BioLegend, San Diego, USA
	CD45RO	BV421	UCHL1	BioLegend, San Diego, USA
	CCR6	PE	11A9	BD Biosciences, Franklin Lakes, USA
B cell panel	CD19	BV605	SJ25C1	BioLegend, San Diego, USA
	CD27	PE	O323	BioLegend, San Diego, USA
	CD38	AF700	HIT2	BioLegend, San Diego, USA
	CD138	FITC	MI15	BioLegend, San Diego, USA
	IgD	PE/Dazzle 594	IA6-2	BioLegend, San Diego, USA
	IgM	PE-Cy7	MHM-88	BioLegend, San Diego, USA
	CD8a	PerCP-Cy5.5	RPA-T8	BioLegend, San Diego, USA
	CD56	PerCP-Cy5.5	HCD56	BioLegend, San Diego, USA
	CD14	PerCP-Cy5.5	HCD14	BioLegend, San Diego, USA
	CD3	PerCP-Cy5.5	OKT3	BioLegend, San Diego, USA
Cytokine panel	CD4	BV510	RPA-T4	BioLegend, San Diego, USA
	CXCR5	APC	J252D4	BioLegend, San Diego, USA
	CXCR3	BV605	G025H7	BioLegend, San Diego, USA
	CCR6	BUV395	11A9	BD Biosciences, Franklin Lakes, USA
	CD45RO	BV650	UCHL1	BioLegend, San Diego, USA
	CD3	PECy7	OKT3	BioLegend, San Diego, USA
	CD19	APC-Cy7	SJ25C1	BioLegend, San Diego, USA
	CD8a	APC-Cy7	RPA-T8	BioLegend, San Diego, USA
	CD56	APC-Cy7	HCD56	BioLegend, San Diego, USA
	CD14	APC-Cy7	HCD14	BioLegend, San Diego, USA
	CD38	AF700	HIT2	BioLegend, San Diego, USA
	IL-2	PerCP-Cy5.5	MQ1-17H12	BioLegend, San Diego, USA
	IL-4	FITC	MP4-25D2	BioLegend, San Diego, USA
	IL-17A	BV785	BL168	BioLegend, San Diego, USA
	IL-21	PE	3A3-N2	BioLegend, San Diego, USA
	IFNg	BV421	4S.B3	BioLegend, San Diego, USA
Tetramer panel	CD4	BV510	RPA-T4	BioLegend, San Diego, USA
	ICOS	FITC	C398.4A	BioLegend, San Diego, USA
	CD3	PE-Cy7	OKT3	BioLegend, San Diego, USA
	CD19	PerCP-Cy5.5	SJ25C1	BioLegend, San Diego, USA
	CD8a	PerCP-Cy5.5	RPA-T8	BioLegend, San Diego, USA
	CD56	PerCP-Cy5.5	HCD56	BioLegend, San Diego, USA
	CD14	PerCP-Cy5.5	HCD14	BioLegend, San Diego, USA
	CXCR5	PE	J252D4	BioLegend, San Diego, USA
	CXCR3	BV605	G025H7	BioLegend, San Diego, USA
	PD-1	BV785	EH12.2H7	BioLegend, San Diego, USA
	CD25	PE/Dazzle 594	M-A251	BioLegend, San Diego, USA
	CD38	AF700	HIT2	BioLegend, San Diego, USA
	CD45RO	BV650	UCHL1	BioLegend, San Diego, USA
	CCR6	BUV395	11A9	BD Biosciences, Franklin Lakes, USA

Supplementary table 2. List of antibodies and staining panels used in the study.
